# Items from the Case-Book

**Published:** 1858-08

**Authors:** J. Y. Wynne

**Affiliations:** Good Hope, Miss.


### ARTICLE IV.

*Items from the Case-Book of* J. Y. Wynne, M. D., of Good Hope, Miss.

In the report of the following cases, the only object in view is the mutual interchange of opinions and modes of practice, which such reports promote.

The reports in Medical Journals of cases which terminate fatally, as well as those in which we are successful, whether the cases are of rare occurrence or not, or whether they are of very great importance from any other consideration, are read with interest by those who have constantly such cases under treatment.

It is to aid, as much as in my power, in keeping up this very useful interchange of thoughts and practice among the members of our whole profession.

*Case 1st.*—A dislocation of the shoulder, of one month's standing, in the person of J. W. B., Esq., came under the treatment of myself and partner, Dr. B. P. Funchess, about the 20th of February. The displacement of the humerus was forward—the head of the bone forming a distinct protuberance just below the clavicle. The luxation occurred in the fall from a horse, doubtless from the weight of the body being received upon the arm while extended toward the back.

The reduction was undertaken, not without some misgivings as to the probabilities of success, and even as to the safety of the operation, after so long a time had elapsed from the receipt of the injury. The patient being brought under complete anasthæsia, by chloroform, and the body fixed by a sheet passed under the arm, traction for not more than five minutes, was sufficient to enable me to restore the head of the bone to its natural position. Some pain succeeded the return of consciousness, but was promptly allayed by morphia. No untoward symptom occurred, and Mr. B. rapidly recovered the use of his arm, which is now as perfect as before the accident.

*Case 2d.*—Jack, a negro man, aged about 30, the property of Mr. H., who, four weeks previously, had been severely hurt by machinery, was placed under my care.

I found him with the following symptoms:—Pulse 120, tongue furred, &c. The injury was received in the pelvis and neighboring bones. I found the toes of one foot turned inwards, the thigh flexed at right angles with the body, with a depression, and no doubt fracture of the pubes. On extending the leg it was ascertained to be three inches shorter than the sound limb. On examination, the femur was found dislocated, upwards and outwards, the head of the bone resting against the dorsum of the ilium. The reduction was undertaken on the 28th of March last, in the usual way, by securing the pelvis by means of a sheet passed over the perineum, and fastened to the wall. Pretty forcible traction upon the injured limb, most of the time for an hour and a half, was necessary to overcome the muscular contraction sufficiently to restore the head of the bone to its proper position.

The patient has so far recovered as to be able to walk, and will, in all probability, regain his usual strength and activity.

*Case 3d.*—Caroline, a colored woman, 35 years of age, the mother of six children, was taken in labor February 10th, at night. The os uteri, which was but slightly dilated, after three hours of considerable uterine contraction, was found rapidly dilating in half an hour after the application of hyoscyamus.

The case proved to be one of twins, with a feet and head presentation, as usual, but in this case the footling first came down and was delivered, the funis, which was of extraordinary length, (five feet) first presenting in advance of the feet. The other child, which was soon after delivered, presented the same anomaly in respect to the length of the cord.

*Case 4th.*—On 23d of April was called, in consultation with Dr. Funchess, to see Chloe, a negro woman, 33 years old, in labor with twins, and received from the Doctor the following statement of the progress of the case previous to my arrival:

Labor commenced early on the night of 22d. The pains, which were at first active, became feeble, and of short duration, when considerable hemorrhage came on. Repeated small doses of Ergot to restore the uterine contractions, arrested the discharge, and the labor progressed favorably to the delivery of one fœtus this morning at 8 o'clock.

The immediate descent and delivery of the second child was, perhaps, prevented by the position of the left arm interfering with the engagement of the head in the pelvis.

In this condition of things, profuse hemorrhage re-occured with convulsions, and consultation demanded.

The last named condition of the uterus, and progress of the labor existed when I arrived. The use of chloroform promptly arrested the convulsions; and in order to deliver the woman more certainly from the danger of protracted labor under existing circumstances, the delivery was hastened by the use of forceps. Both children and the mother finally did very well.

